# Effect of co-flow on fluid dynamics of a cough jet with implications in spread of COVID-19

**DOI:** 10.1063/5.0064104

**Published:** 2021-10-12

**Authors:** Sachidananda Behera, Rajneesh Bhardwaj, Amit Agrawal

**Affiliations:** Department of Mechanical Engineering, Indian Institute of Technology Bombay, Mumbai 400076, India

## Abstract

We discuss the temporal evolution of a cough jet of an infected subject in the context of the spread of COVID-19. Computations were carried out using large eddy simulation, and, in particular, the effect of the co-flow (5% and 10% of maximum cough velocity) on the evolution of the jet was quantified. The Reynolds number (Re) of the cough jet, based on the mouth opening diameter (*D*) and the average cough velocity, is 13 002. The time-varying inlet velocity profile of the cough jet is represented as a combination of gamma-probability-distribution functions. Simulations reveal the detailed structure of cough jet with and without a co-flow for the first time, to the best of our knowledge. The cough jet temporal evolution is similar to that of a continuous free-jet and follows the same routes of instability, as documented for a free-jet. The convection velocity of the cough jet decays with time and distance, following a power-law variation. The cough jet is observed to travel a distance of approximately 1.1 m in half a second. However, in the presence of 10% co-flow, the cough jet travels faster and covers the similar distance in just 0.33 s. Therefore, in the presence of a co-flow, the probability of transmission of COVID-19 by airborne droplets and droplet nuclei increases, since they can travel a larger distance. The cough jet without the co-flow corresponds to a larger volume content compared to that with the co-flow and spreads more within the same range of distance. These simulations are significant as they help to reveal the intricate structure of the cough jet and show that the presence of a co-flow can significantly augment the risk of infection of COVID-19.

## I. INTRODUCTION

As of July 2021, the outbreak of coronavirus-based COVID-19 disease has claimed more than 4.06 million lives while 188 million people have been infected by the disease worldwide. Respiratory activities, like talking, breathing, sneezing, and coughing, generate respiratory aerosol droplets, which seem to be the dominant mode of transmission of the COVID-19 disease^1–3^ as they carry infectious pathogens. The distance traveled by these aerosol particles containing pathogens depends upon the particle size as well as the velocity with which they are expelled.^4^ Zayas *et al.*^5^ suggested that around 99% of the aerosols generated during coughing are less than 10 *μ*m in size. Understanding the transmission of these aerosol particles containing pathogens is essential for mitigating the spread of the pandemic.^6^

The literature suggests that there are three possible ways for transmission of the virus between an infectious subject and a healthy subject:^6–8^ *contact transmission*, *large droplet transmission*, and *airborne transmission*. In the case of *contact transmission* and *large droplet transmission*, a susceptible person could get infected by coming in direct contact with a contaminated surface or by physically inhaling the droplets. The contact transmission has been studied extensively in the past, where the survival of the virus on various types of surfaces has been analyzed.^9–13^ In cases of *airborne transmission*, the droplet nuclei or the smaller size droplets can travel longer distances with ambient air.^14^ Wei and Li^15^ through their computational study predicted the spread and trajectory of particles of various sizes and showed that the smaller size droplets could travel long distances (of around 4 m). The droplets generated from human cough can travel up to a distance of 2 m in a static background.^16^ However, with the ambient wind speed in the range of 4–15 km/h, the droplets can travel an even longer distance (around 6 m).^14,17^ Bourouiba, Dehandschoewercker, and Bush^6^ and Bourouiba^18^ suggested that the spread and trajectory of the droplet particles strongly depend on the strength of the cough or sneeze, the ambient flow speed, and the turbulence. They also suggested that the smaller size droplets (<5–10 *μ*m) always follow the turbulent cough jet. Wang *et al.*^19^ studied the effect of temperature and humidity on the droplet airborne lifetime and suggested that 50 *μ*m droplets have large range of airborne lifetime compared to 100 *μ*m droplets. The bigger size droplets (50–200 *μ*m) are significantly affected by gravity and inertia force and fall with the weakening of the flow field.^20,21^ However, the smaller size droplets (<30 *μ*m) are least affected by gravity or inertia.^20^ Therefore, their trajectory is significantly affected by the ambient flow. From the above discussion, it is concluded that the smaller droplet particles containing infectious pathogens have a longer residence time in air and can travel longer distance. Also these small droplets are least affected by gravity or inertia and merely follow the ambient air flow. Such information is highly useful because it helps in taking proper measures to reduce the probability of spread of infection in a community.

The transient velocity distribution and direction of cough flow have been experimentally studied using particle image velocimetry.^20,22,23^ Mahajan *et al.*^24^ suggested that the peak velocity time for coughing is in milliseconds, and to capture the cough jet velocity and distribution, it is essential to have a high-frequency measurement system. Most of the earlier studies were performed with time intervals between 67 and 270 ms, which is too long to characterize a cough flow velocity field. Gupta *et al.*^25^ advocated that measurements of the flow rate and velocity distribution should be performed with a frequency of 100 Hz or higher. The maximum velocity of a cough flow is in the range of 6–28 m/s. Using Schlieren technique, Tang *et al.*^26^ calculated the maximum cough velocity to be 8 m/s. The velocity distribution of a cough is found to be complex,^25,27^ and Gupta *et al.*^25^ suggested that the flow rate variation in the case of cough with time can be represented as a combination of two different gamma-probability-distribution functions. Many numerical simulations have been performed in the past where the dispersion of the cough droplet particles in the indoor environment have been studied.^28–32^ In general, a cough jet is characterized by a leading vortex puff with a trailing flow.^33^ The vortex structure in the case of cough flow may play an important role in the transport of droplet particles.^34^ The complex coughing phenomenon is sometimes approximated as continuous or a steady flow of jet.^4,15^ Rim^35^ showed that the particles in the case of transient cough jet, where the particles are released for a shorter duration, have a lower exposure compared to the particles continuously released in the case of steady jet. Therefore, the duration of coughing plays a major role in determining the penetration and transport of the cough droplets.

In general, the duration of coughing is around 0.5–0.6 s during which around 0.6–1.6 L airflow is produced.^23,25^ Generally a cough flow is expected to show the property of a continuous jet/plume (initially) and interrupted jet (afterward). Therefore, the theories of classical fluid mechanics on jet/plumes and interrupted jet can be useful in providing insight into the development of a cough flow.^35–40^ Most of the existing studies (barring a few) modeled the cough flow with a simple temporal exit velocity, like a simple puff (which is a sudden release of a finite amount of fluid)^23^ or a simple pulsating profile. The flow dynamics of a simple puff has been discussed earlier in the literature.^41–43^ A real cough exhibits, as discussed earlier, a very complex temporal velocity distribution and is approximated as a combination of a gamma-probability-distribution function.^25^ Our objective is to perform a large eddy simulation (LES) of the cough jet flow using the complex velocity profile suggested by Gupta *et al.*^25^ and Dudalski *et al.*^27^ Further, it is now established that the very large size virus-laden droplets (diameter of the order of mm) are not affected by the exhaled air during the coughing and travel semi-ballistically before falling down rapidly due to the gravitational pull. The smaller size droplets (diameter ≲ 10 μm) are generally suspended in the cough jet and advect with it. These small size droplets, as they advect with the cough jet, are also subjected to gravitational settling and evaporation. The droplets with settling speed smaller than the surrounding cough jet remain trapped longer within the cough jet. Due to the continuous evaporation of the cough droplets, the water content of the droplets decreases and the droplets become droplet nuclei. These droplet nuclei have very small settling speed and, thus, remain trapped in the cough jet and are advected with it. Thus, their transmission will largely depend on the behavior of the underlying cough jet.

Therefore, understanding the nature of evolution of cough jet is essential for a good understanding of the dispersion of droplets of various sizes. Also as the cough jet evolves in the streamwise direction, the volume content of the cough jet increases due to entrainment of surrounding air.^44,45^ The distance that a cough jet would travel and the volume of contaminated air within it are important parameters to determine. Moreover, most of the previous computational work analyzed the coughing into a static environment. However, in reality, there is always a background flow (although its velocity could be small) escorting a cough. Depending on the wind speed, the surrounding environment of a cough-jet could be divided into various categories.^46^ Generally, a wind speed below 0.45 m/s is considered as a calm environment. The wind speed in indoor conditions is generally low and ranges between 0.04 and 0.3 m/s.^47^ Hence, coughing in indoor conditions can be analyzed by modeling it as a cough jet in a static environment. The wind speed in the range of 0.45–1.34 m/s is categorized as light air, and the wind speed between 1.8 and 3.1 m/s is defined as light breeze. In the case of a gentle breeze, the wind speed is in the range of 3.5–5.4 m/s. The wind speed in the outdoor condition varies throughout and is generally in the range of 0.45–5.4 m/s.^46^ These background flow velocity may play an important role in the transmission of the droplets, particularly the droplet-nuclei that remains trapped in the cough jet and convect with it.

Motivated by these issues, the present study investigates the temporal evolution of the flow structures associated with a cough jet. Toward modeling the coughing into a background, which is not static, the cough jet is simulated with the co-flow and the streamwise penetration distance of the cough jet with the co-flow is analyzed. The effect of the co-flow in the direction of the cough is only considered in the present study. The effect of a counter flow or a cross-flow (which may be possible scenario) on the cough jet is not considered in the present study. The specific aims of the work are to compute the distance traveled and volume contained in a cough jet, with and without a co-flow, and to examine the underlying flow structure, which is responsible for the observed behavior. A second objective is to use this information to understand the implications of the spread of COVID-19 by an infected cough jet in a realistic scenario (with a co-flow).

## II. METHODOLOGY

### A. Large eddy simulation

The present study employs a Large eddy simulation (LES) method to simulate the cough jet. The cough jets in general have a Reynolds number of the order of 10^4^ and are found to be turbulent in nature.^27,48^ Turbulent flows are associated with a wide range of scales, both temporal and spatial. The smallest of length scales in a turbulent flow are the Kolmogorov length scales (at which dissipation occurs), while the largest length scales could be as big as the characteristic length of the flow domain. Carrying out the computation of the turbulent flows is relatively difficult and expensive in terms of computational resources and central processing unit (CPU) time, particularly direct numerical simulation (DNS). The computational cost associated with DNS studies is high and increases with an increase in the Reynolds number, as in a DNS study, resolution of all scales all the way up to the dissipation range is required. A popular alternative to the DNS study of turbulent flows is large eddy simulation. The principal idea behind LES is to use a low-pass filtering operation, where the smaller length scales that are computationally more expensive to resolve are separated from the larger scales, and only the larger scales are resolved by the computational grid employed. The effect of the smaller scales on the flow field is then suitably modeled. The filtered governing equations for LES are

$$\frac{\partial {\tilde{u}}_{i}}{\partial x_{i}}=0,$$ (1)

$$\frac{\partial {\tilde{u}}_{i}}{\partial t}+{\tilde{u}}_{j}\frac{\partial {\tilde{u}}_{i}}{\partial x_{j}}=-\frac{\partial \tilde{p}}{\partial x_{i}}+\frac{1}{Re}\frac{\partial^{2}{\tilde{u}}_{i}}{\partial x_{j}^{2}}-\frac{\partial \tau_{ij}}{\partial x_{j}},$$ (2)

where $\tau_{ij}=\tilde{u_{i}u_{j}}-\tilde{u_{i}}{\tilde{u}}_{j}$ is the SGS (subgrid-scale) stress tensor. In the present study, the SGS stress tensor is modeled using a “One equation eddy-viscosity SGS model (OEESM)”^49^ and the anisotropic part of the SGS term is given as

$$\tau_{ij}=\frac{1}{3}\delta_{ij}\tau_{kk}-2\nu_{T}{\tilde{S}}_{ij}.$$ (3)

The subgrid scale eddy viscosity *ν_T_* is computed using *k_sgs_*, where *ν_T_* is defined as

$$\nu_{T}=C_{k}\sqrt{k_{\mathrm{sgs}}}\Delta .$$ (4)

**FIG. 1.**
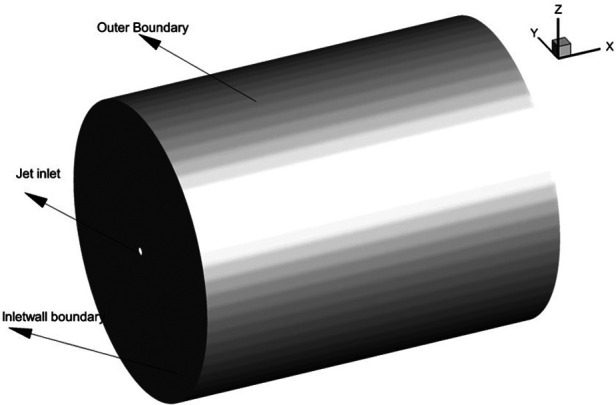
Computational domain.

*C_k_* is model constant with a default value of 0.094. The difference between Smagorinsky SGS model and the OEESM model is the way that the subgrid scale kinetic energy (*k_sgs_*) is computed. The Smagorinsky model assumes a local balance between the SGS energy production and dissipation, but the OEESM model solves a transport equation for *k_sgs_*. The open-source Computational Fluid Dynamics code (OpenFoam^50^) was used to solve the governing equation. The finite volume discretization method on a structured grid arrangement was employed for the computations. For both the spatial and temporal terms, second order accurate discretization schemes were employed. The computational domain used in the present study is shown in Fig. 1. The streamwise extent of the computational domain is 1.085 m, and all the analysis of the cough jet in the present study is done within this domain of interest.

**FIG. 2.**
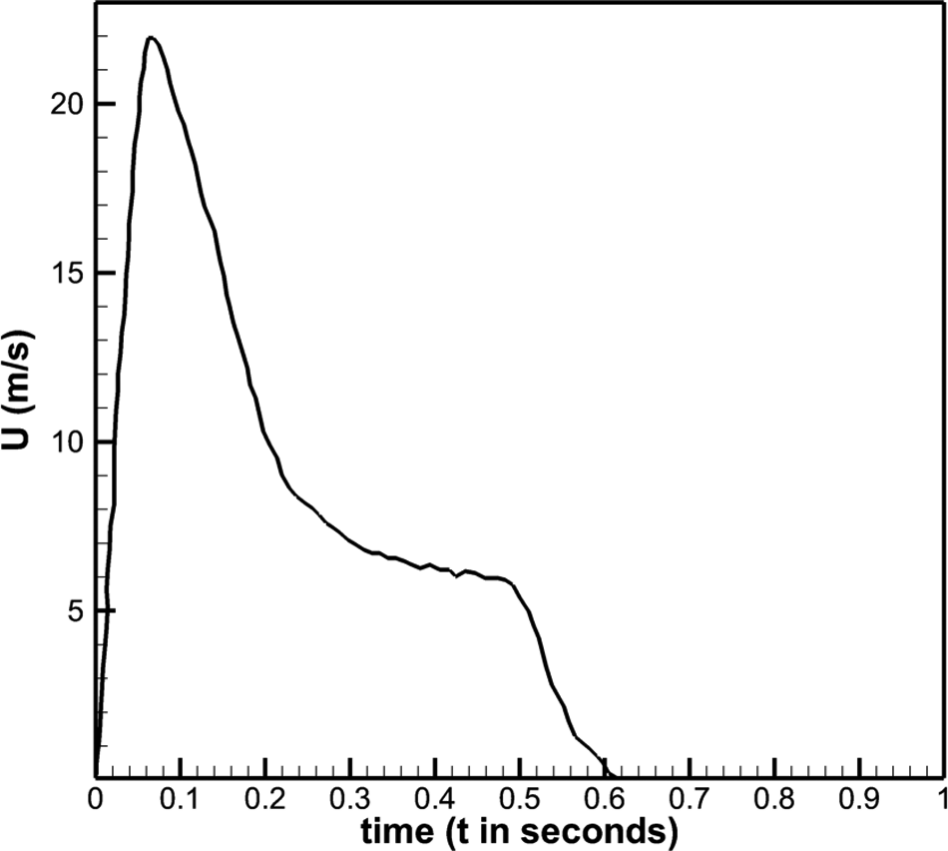
Velocity profile reported in Dudalski *et al.*^27^ is specified at the jet inlet in the present work.

At the inlet of the jet, the velocity profile used is based on the average volumetric flow rate at the mouth exit based on the average mouth opening diameter.^25,27^ The jet inlet velocity specified in the present simulation is the same as that in the study of Dudalski *et al.*^27^ and used the mouth opening diameter as *D *=* *0.0217 m. The specified velocity profile is shown in Fig. 2. The duration of the coughing processing is typically around 0.4–0.6 s.^51^ In the present study, the duration of the coughing process is considered to be 0.61 s. The average cough velocity for the entire duration of the coughing process in the present study is taken to be 8.808 m/s. Therefore, the average Reynolds number for the coughing process in the present study is calculated to be approximately Re ≊ 13002. The present study simulates the cough jet with and without the co-flow. For the cough jet without a co-flow, the velocity at the inlet boundary of the computational domain is specified as zero ( u=v=w=0), except for the jet inlet (refer to Fig. 1). For the cough jet with a co-flow, the streamwise velocity at the inlet boundary is specified as $u=0.05u_{\mathrm{jet},\text{max}}$ (for 5% co-flow) and $u=0.1u_{\mathrm{jet},\text{max}}$ (for 10% co-flow), while the rest of the velocity components are specified as zero ( v=w=0). As discussed in Sec. I, the wind speed in outdoor conditions is generally between 0.45 and 5.4 m/s,^46^ and in the indoor condition, the wind speed is between 0.04 and 0.3 m/s.^47^ Therefore, in the present study, the co-flow with 5% and 10% that makes an approximate velocity of 1.1 and 2.2 m/s, respectively, is similar to the speed encountered in outdoor conditions. The cough jet without a co-flow can be thought of a coughing into a generally calm environment.

### B. Data post-processing

The structure of the jet is gleaned by studying the vortical structures present in the flow field. To identify the vortical structures in the cough jet, the vortex identification technique Q-criterion^52^ is used. The Q-criterion is mathematically defined as $Q=\frac{1}{2}(\Omega_{ij}\Omega_{ij}-S_{ij}S_{ij})$. Here, $\Omega_{ij}=(u_{i,j}-u_{j,i})/2$ and $S_{ij}=(u_{i,j}+u_{j,i})/2$ are, respectively, the anti-symmetric and symmetric components of the velocity gradient tensor ▽u. Positive Q iso-surface defines vortical structures as it isolates the area where the strength of rotation is greater than the strength of the strain rate. Since vorticity increases as the center of the vortex is approached, Q can be expected to remain positive in the core of the vortex. The positive Q regions are found to be good indicators of the coherent vortices in various wall-bounded and free-shear flows.

The volume content of the cough jet is an important parameter in the study of the transmission of COVID-19 through coughing. The volume content of the cough jet is the volume bounded by the boundary^53,54^ of the cough jet. The boundary or interface between the cough jet, which is turbulent in nature and its surrounding, which is non-turbulent, is calculated by the method proposed by Bisset *et al.*^53^ In this method, the magnitude of vorticity vector with a suitable threshold value is used to detect the interface or the boundary between the cough jet and its non-turbulent surrounding. Once the boundary of the cough jet was found out, the volume was calculated by adding of the volume of all the cells inside the boundary.

## III. RESULTS AND DISCUSSION

### A. Temporal evolution of cough jet

**FIG. 3.**
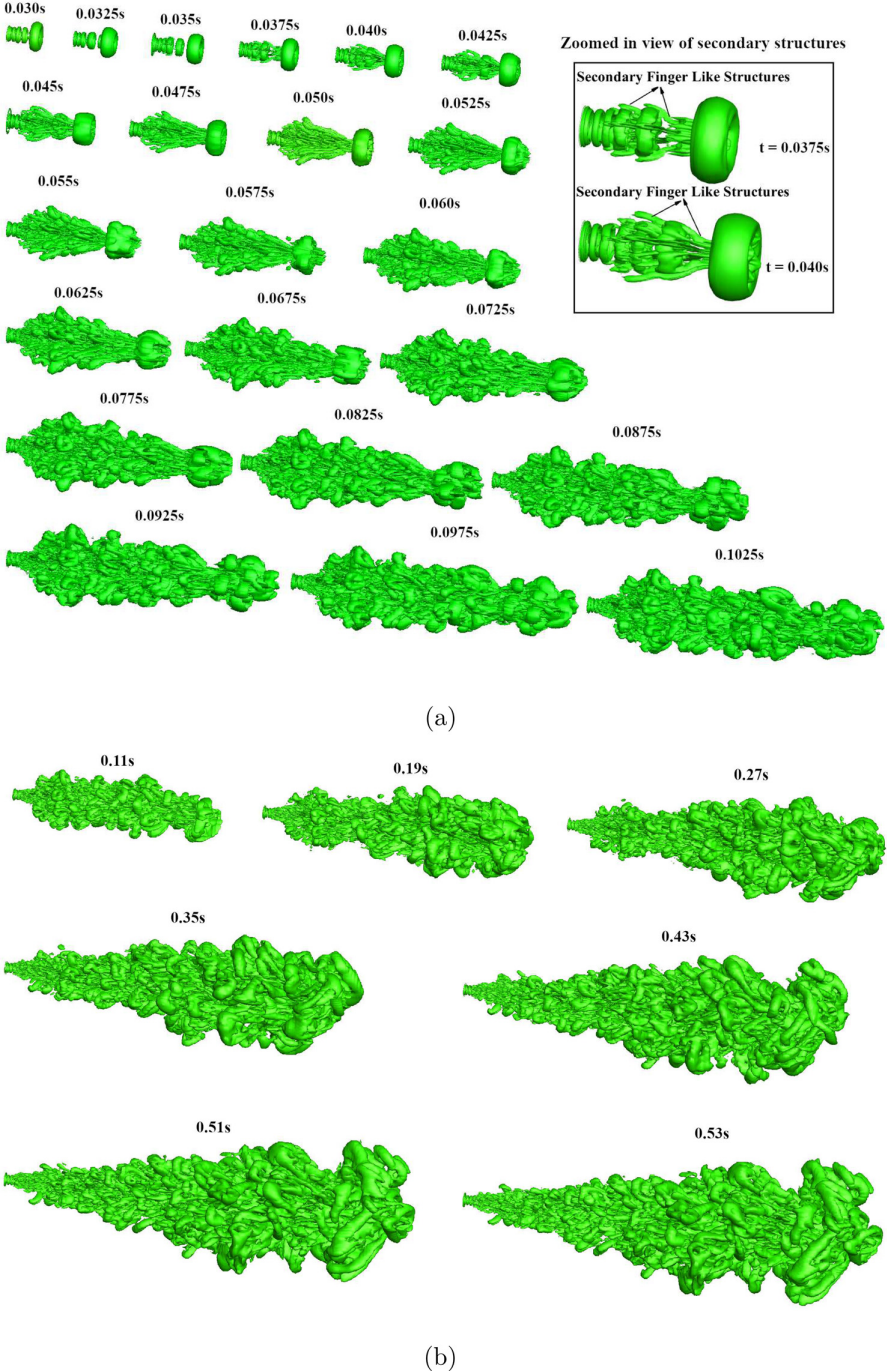
Iso-surface of Q criterion representing evolution of cough jet without the co-flow: (a) *t *=* *0.03–0.1025 s and (b) *t *=* *0.11–0.53 s. The flow is from left to right, and the time stamps represent the simulation time in seconds. The inset shows the finger-like structures formed by flow instability. Multimedia view: https://doi.org/10.1063/5.0064104.110.1063/5.0064104.1

The evolution dynamics of the cough jet after coming out of the orifice, with and without the co-flow, is presented in this section. The iso-surface of the Q-criterion showing the temporal evolution of the vortical structures associated with the cough jet without the co-flow is plotted in Fig. 3 (Multimedia view). The examination of the temporal evolution of the vortical structures shows only a train of ring-like structures in the initial region of the jet due to shear layer instability (*t *=* *0.030–0.035 s). Simha and Rao^55^ pointed out the important role that vortex rings play while performing experiments with subjects coughing into still ambient. They reported periodic ejection of vortex rings and conjectured that the vortex rings are formed by the vibration of the vocal cords. It is difficult to resolve the origin in the experiments, and our simulations suggest a fluid dynamics origin brought about by shear layer instability behind the formation of the vortex ring.

Liepmann and Gharib^56^ studied the pairing of the vortex rings occurring in the initial jet region of a continuous jet. They suggested that if two upstream structures are close enough to each other, or one of the vortex rings is relatively small, then the vortices or the rings will merge. Similar observations are also made in the near field of cough jet in the present study. The pairing of the vortex rings in the near field of the jet exit can be observed for all the time instances from Fig. 3(a). The pairing or merging of the vortex rings continues in the initial region, and the resultant vortex ring grows in size and convects downstream. At *t *=* *0.035 s, a train of axisymmetric vortex rings is observed in the jet region. However, at *t *=* *0.0375 s, the primary flow structures seem to become azimuthally unstable creating secondary instability. The secondary instability takes the shape of “finger”-like structures that develop from the braid region between two vortex rings [shown in the inset of Fig. 3(a)], stretch around the following vortex ring. Gohil *et al.*^57^ have also reported similar “finger”-like secondary structures for a continuous circular free-jet at *Re *=* *10 000. Their LES study revealed multiple single vortex pairing events along with the tearing of structures into one or more structures.

Although vortex pairing is observed in the present study, the tearing of the structures into one or more structures is found to be absent. The secondary flow structures developed from the braid region between two vortex rings stretch and grow with time and interact with the upstream vortex rings. Subsequently, stronger non-linear interaction between the primary and secondary structures leads to hairpin-like structures. The presence of the hairpin type structures are also reported by Gohil *et al.*^57^ in the far field of a circular jet. It is also noted that the vortex rings in the far field that describes the cough wave-front (also called leading vortex ring) become azimuthally unstable at a later time instance (*t *=* *0.055 s) compared to the vortex rings that were following it. The significance of the leading vortex ring was highlighted by Renzi and Clarke,^58^ who suggested that the leading vortex ring is responsible for the large range of the droplet movements. They showed that the leading vortex ring, which is relatively a large and strong vortex, serves to re-suspend the droplets as the droplets settle out of the cloud due to their weight. They also highlighted that the smaller size droplets are influenced the most by the leading vortex and follow the trajectory of the leading vortex ring closely. By *t *=* *0.0725 s, the leading vortex ring defining the cough wave-front starts to break down due to the strong azimuthal instability and gradually gives rise to very complex flow structures.

The evolution process of the cough jet continues with time becoming more complex in the streamwise direction [refer Fig. 3(b)]. In the present study, the evolution of the cough till the time the cough wave-front reaches the exit of the domain is only simulated. The classical arrow-head shaped structures reported in continuous jets^59^ do not seem consistent with the present flow. It must be noted that similar to a continuous jet the cough jet also spreads radially due to entrainment of the ambient fluid, consistent with the observation of Agrawal and Bhardwaj.^44^

### B. Effect of co-flow on the characteristics of cough jet

**FIG. 4.**
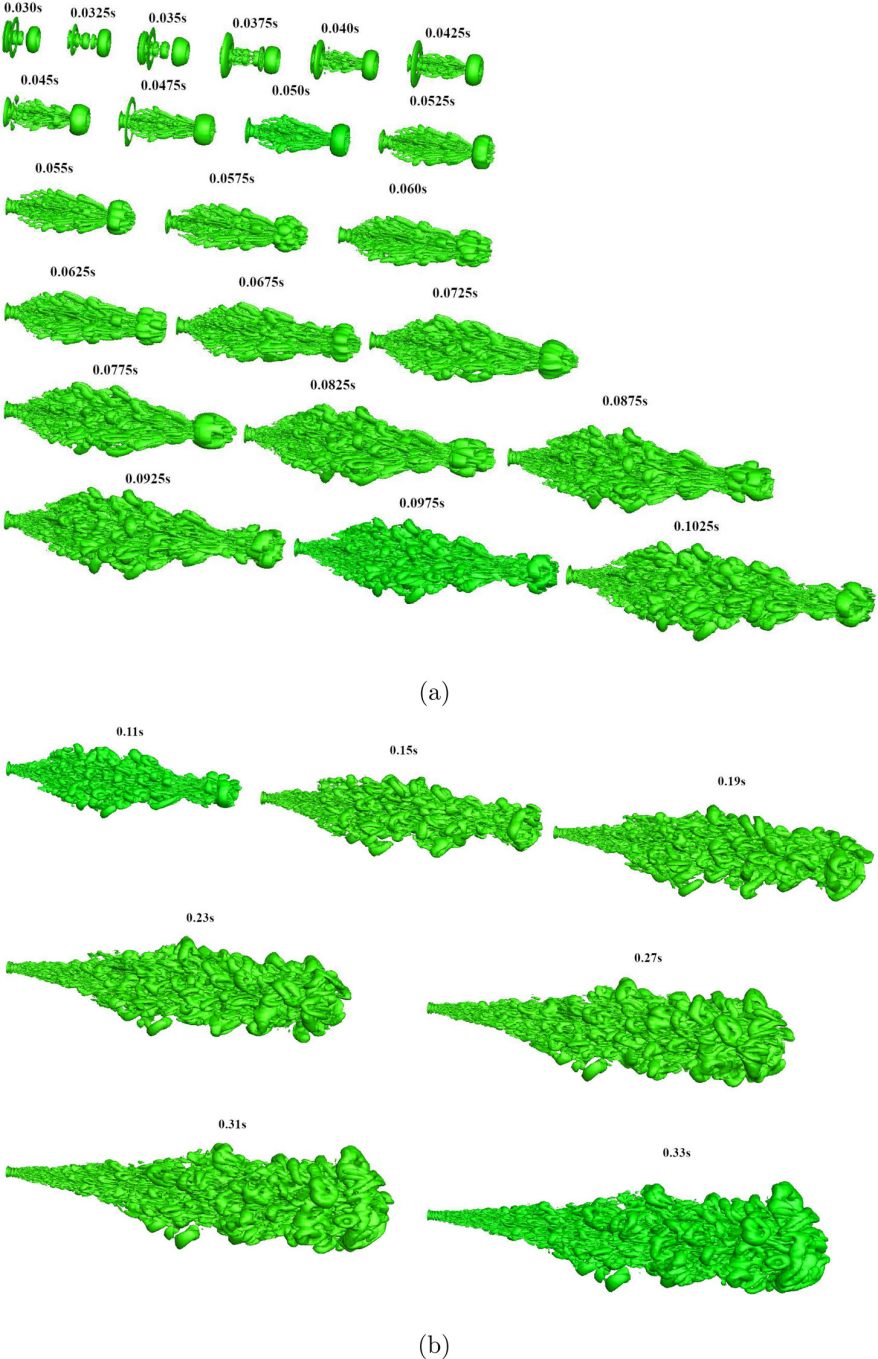
Iso-surface of Q criterion representing evolution of cough jet with 10% co-flow: (a) *t *=* *0.03–0.1025 s and (b) *t *=* *0.11–0.33 s. The flow is from left to right, and the stamps represent the simulation time in seconds. Multimedia view: https://doi.org/10.1063/5.0064104.210.1063/5.0064104.2; https://drive.google.com/file/d/1uT5SLnqURj7jQT0NSiHJhJEf_pxiwGn-/view?usp=sharing.

The evolution of the cough jet with 10% co-flow, shown in Fig. 4 (Multimedia view) reveals similar flow features as that of the cough jet without the co-flow. Similar to the cough jet without the co-flow, the cough jet with the co-flow also shows the formation of a train of vortex rings close to the jet initial region. Qualitatively, the flow structures are similar for a cough jet with and without the co-flow and are evident from the present study with major difference being that the jet without the co-flow seems to have a higher radial spreading. Also the cough wave-front in the case of the cough jet with the co-flow seems to move faster, as discussed next.

**FIG. 5.**
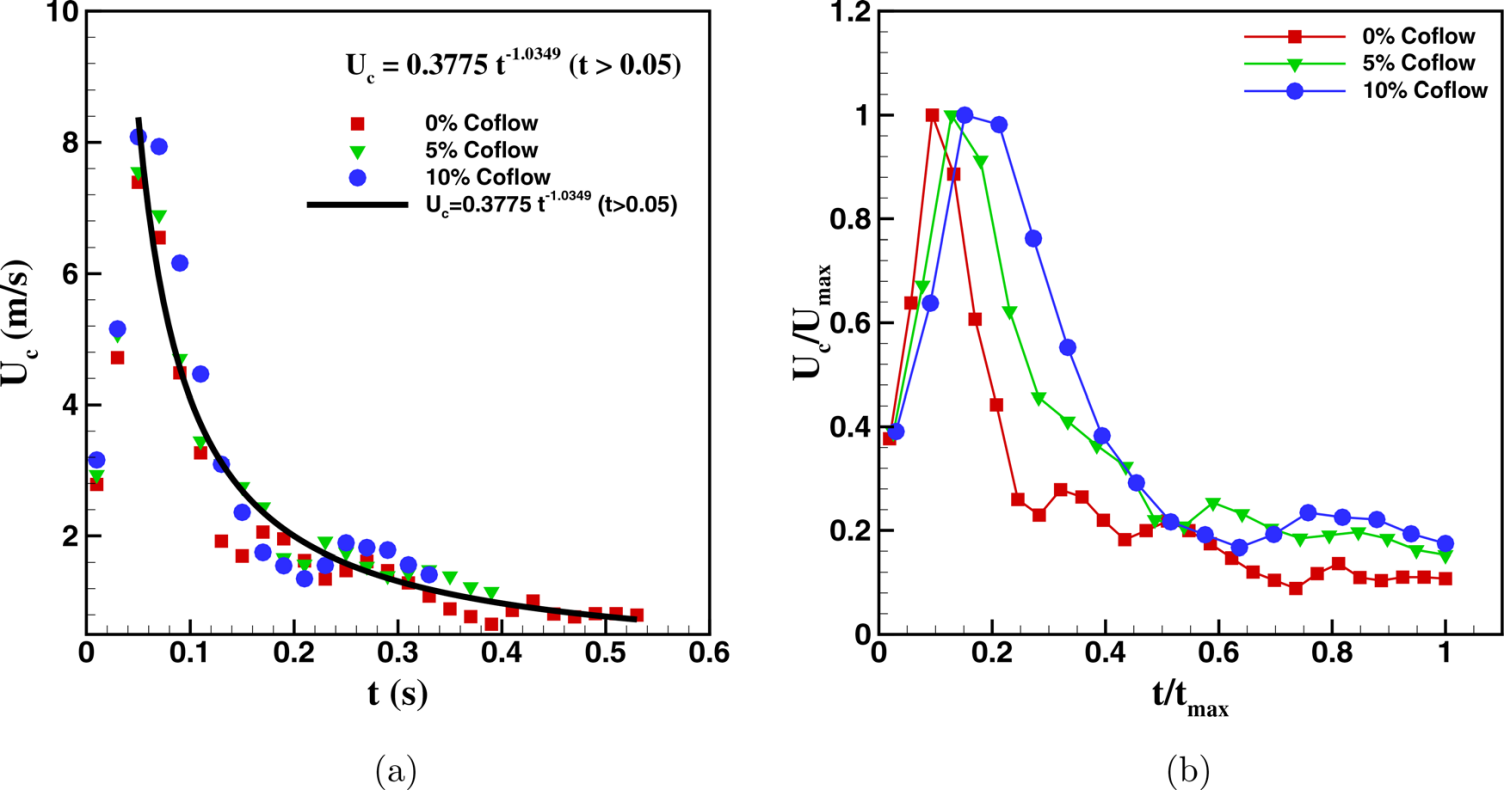
(a) The convection velocity of the cough as a function of time with and without co-flow. (b) The normalized distribution of the convection velocity of the cough with time.

The convection velocity (*U_c_*) of the cough jet as a function of time is plotted in Fig. 5. To evaluate the convection velocity of the cough jet, distance (*s*) traveled by the cough with time is noted. Here, *s* represents the instantaneous position of the cough front. The convection velocity of the cough is then evaluated as $U_{c}=\frac{ds}{dt}$ using the central difference scheme. The convection velocity of the cough jet, with different percentage of co-flows, plotted in Fig. 5(a), shows that convection velocity reaches a maximum value and then gradually decreases with time *t* for all the cases. A single curve is fitted for the decaying region of the convection velocity and time profile for all the cases of the co-flow and is given by the expression

$$U_{c}=0.3775t^{-1.0349}\;\text{for}\;t>t_{\mathrm{peak}}.$$ (5)

In this context, Wang *et al.*^60^ for their experimental study of coughing also deduced a similar relationship between the convection velocity *U_c_* and time *t*. They suggested that *U_c_* holds a constant value of 6.48 m/s for $t<t_{\mathrm{peak}}$ and then decreases as $U_{c}=0.3t^{-0.7}$ for $t\geq t_{\mathrm{peak}}$. The faster decay in the present study compared to Wang *et al.*^60^ could be due to the difference in the flow condition. In the present study, the coughing is considered as a single expulsion event. However, in the study of Wang *et al.,*^60^ the coughing is suggested to be pulse-like jet, suggesting that in their study, the coughing may actually be a multiple expulsion event. Figure 5(a) also highlights that the cough jet with the highest percentage of the co-flow has the maximum peak convection velocity and the cough jet with no co-flow has the minimum peak convection velocity associated with it. The normalized distribution of the convection velocity of the cough jet with time is plotted in Fig. 5(b). The convection velocity of the cough jet is normalized by the peak convection velocity of the cough (*U_max_*), and the time is normalized by the time taken by the cough jet to reach the exit of the domain (*t_max_*). The normalized plot reveals that the cough jet with higher co-flow travels faster with time due to its higher convection velocity. The convection velocity of the cough jet with the co-flow of 10% is found to be 4.01 m/s, while that of the cough jet with the co-flow of 5% and 0% (no co-flow) is found to be 2.771 and 2.034 m/s, respectively. The higher convection velocity of the cough jet with the co-flow reconfirms that the cough jet with higher co-flow travels faster.

**FIG. 6.**
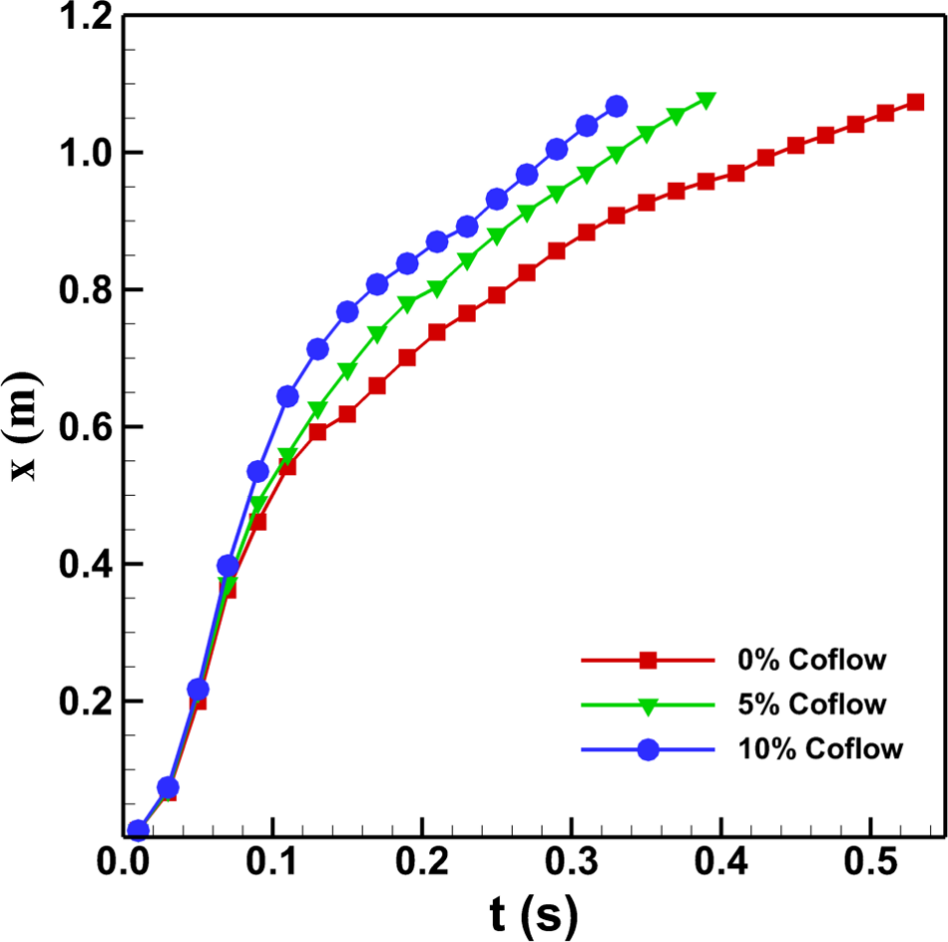
Streamwise penetration distance of the cough jet with and without co-flow as a function of time.

Figure 6 shows the streamwise penetration distance of the cough jet as a function of time with and without the co-flow. It is observed that the cough jet with and without a co-flow penetrates almost similar distances up to time *t *=* *0.05 s. However, after *t *=* *0.05 s, the cough jet with higher co-flow travels faster and penetrates or covers a larger distance with time compared to the cough jet with a lower co-flow or no co-flow. It is noted that the cough jet without the co-flow covers an approximate length of 1.1 m in half a second. While the cough jet with the co-flow of 10% takes around 0.33 s to cover the same distance. The cough jet with the co-flow of 5% lies in between these two cases. The result suggests that cough jet without the co-flow experiences a greater resistance from the ambient surrounding and, hence, penetrates through a smaller streamwise distance compared to the cough jet with a co-flow.

**FIG. 7.**
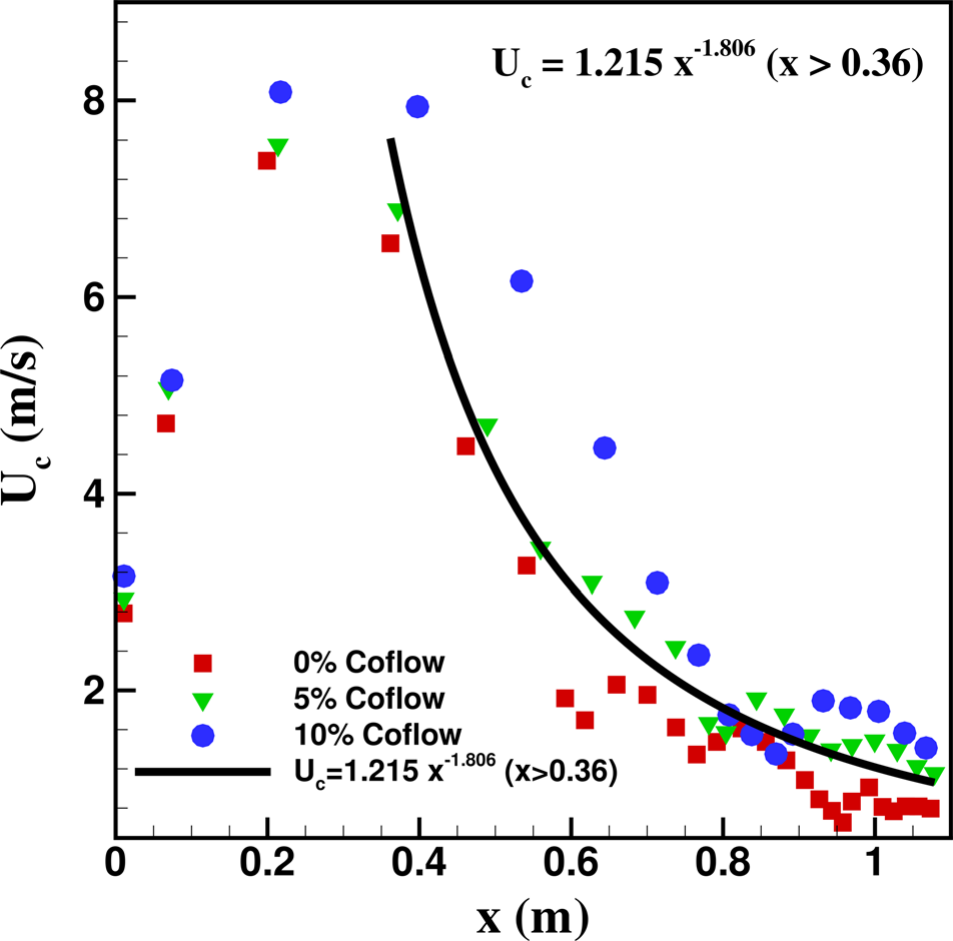
Distance-velocity profile of the cough jet.

The velocity of the cough wave-front with streamwise distance is plotted in Fig. 7 and shows a distribution similar to that of the velocity-time distribution. As the cough jet develops in the streamwise direction, the velocity associated with the cough wave-front is seen to increase up to the streamwise location *x *=* *0.36 m beyond which the velocity decays with streamwise distance. The best fit for the velocity-distance profile in the velocity decaying zone is found to be the power-law fit given by the expression

$$U_{c}=1.215x^{-1.806}.$$ (6)

### C. Implications to spread of COVID-19

Balachandar *et al.*^61^ suggested that the advection of the airborne droplets and droplet nuclei trapped in the cough jet increases the risk of transmission of COVID-19 disease with time, as a distance longer than expected can be covered. This is because if the motion of the cough jet containing droplets is ignored, then the airborne droplets and droplet nuclei will be subjected to a relatively large drag and the droplets will not be able to transmit more than a few centimeters. Following the discussion of Balachandar *et al.*^61^ and the present finding, which shows a longer penetration distance of the cough jet with a co-flow, it can be inferred that with the co-flow the transmission of the virus laden droplet nuclei has the higher probability of transmitting longer distance compared to that without the co-flow in a similar time period.

**FIG. 8.**
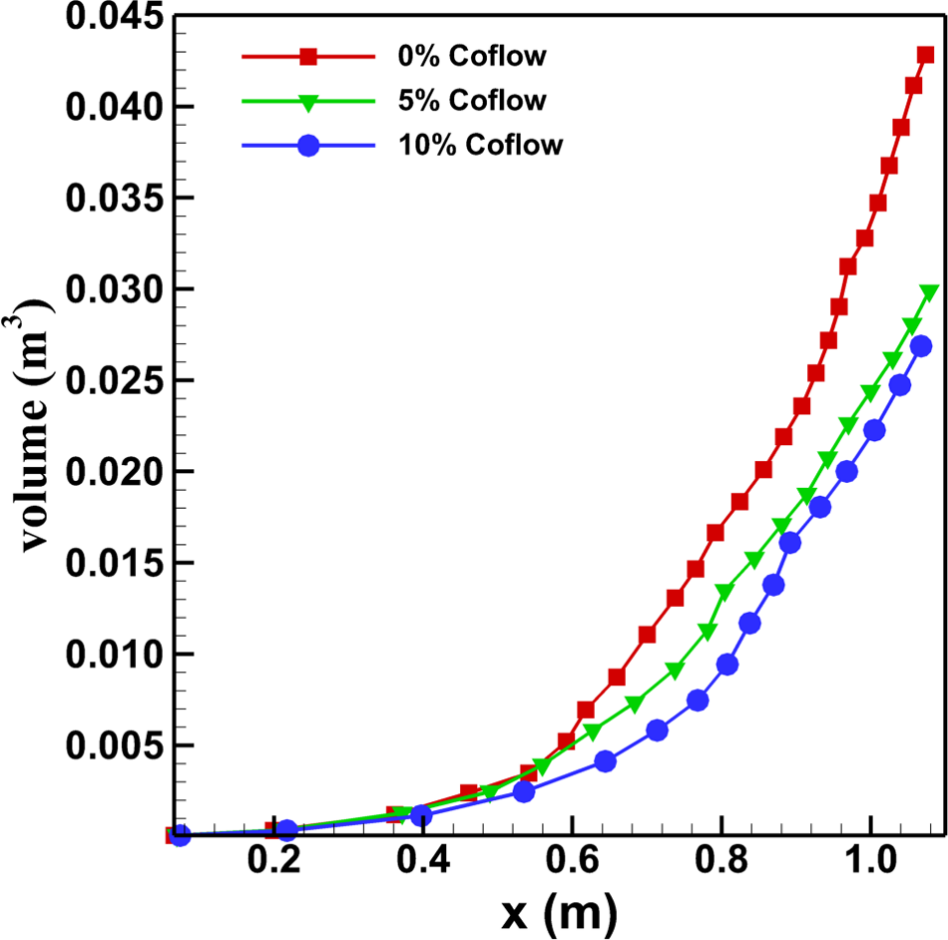
Distribution of volume content of the cough jet with streamwise distance.

The volume content of the jet with its evolution in the streamwise direction is shown in Fig. 8. It is observed that the volume of the cough jet increases with distance as the jet evolves in the streamwise direction. At any particular streamwise location (except near the jet exit region), the cough jet without the co-flow has a higher volume content compared to the cases with the co-flow. For time *t *<* *0.05 s, the cough-jet with and without co-flows almost penetrates similar distances during which its volume content and velocity are almost similar. This may be due to the fact that during the initial time period *t *=* *0.0375 s, the primary vortex rings seem to dominate the flow field of a cough jet and seem to be least affected by the co-flow. With time as the secondary instability initiates (*t *=* *0.04 s), grows, and dominates (*t *=* *0.05 s), the flow field of the cough jet is then found to be affected by the presence of the co-flow. The volume content of the cough without the co-flow near to the exit of the computational domain is around 42.83 l, while with the co-flow of 10%, it reduces to around 26.87 l. Agrawal and Bhardwaj^44^ suggested that a cough (without a co-flow) with a starting velocity of 6 m/s traveling a distance of 1.5 m will approximately have a volume content of 23.5 l. However, the present study of cough jet without the co-flow having an average velocity of 8.808 m/s and traveling a distance of 1.07 m has a volume content of 42.83 l, almost double of the volume content reported by Agrawal and Bhardwaj.^44^ The difference in the volume content between the two studies is attributed to the difference in the cough expired volume (CEV). Agrawal and Bhardwaj^44^ assumed that the volume of air exhaled by a person during coughing is 1 l. However, in the present study, the value of CEV is approximately 1.98 l, which explains the difference in the volume content with that in Agrawal and Bhardwaj.^44^ While the CEV considered in the present study is higher than Agrawal and Bhardwaj^44^ (almost twice), it is found to be in agreement with some of the earlier studies. Gupta, Lin, and Chen^25^ suggested that the CEV for male subjects varies between 0.4 and 1.6 l, while in the case of females, it varies between 0.25 and 1.25 l. Similarly Ren *et al.*^51^ reported the average CEV for 700 subjects to be 1.54 l.

The lower volume of the cough jet with the co-flow is attributed to a smaller lateral spread of the jet in the presence of a co-flow. As explained in Agrawal and Bhardwaj,^45^ the probability of infection is expected to be higher in cough jet with the co-flow as the flow experiences lesser dilution, due to reduced amount of entrainment of ambient air in the cough jet. Having a co-flow can, therefore, have adverse effect in two ways: the cough jet travels faster and dilutes the contaminated air lesser, as compared to cough jet without the co-flow. Therefore, in outdoor conditions, it is highly suggested to always wear a mask as the background breeze can propagate the virus faster to a longer distance without diluting it and, hence, increases the chances of infection.

## IV. CONCLUSIONS

The temporal characteristic of a cough jet with and without co-flows was examined numerically using large eddy simulation in this work. This is one of the first works employing LES for studying cough jet, especially in the presence of a co-flow. Two different amounts of the co-flow (5% and 10% of the maximum cough velocity) are examined in this work.

The temporal evolution of cough jet with and without co-flows is found to be similar to that of a continuous free-jet, where a train of ring vortex structures are observed in the jet exit region due to jet shear layer instability. The phenomenon of vortex pairing along with the secondary instability in the form of finger-like structures was also observed similar to a continuous free-jet. The convection velocity of the cough jet seems to decay with distance due to entrainment of ambient air and the decaying occurs following a power law. The cough jet without the co-flow has a lower axial velocity and higher expansion rate compared to a cough jet with the co-flow. The higher volume content of the cough jet without the co-flow also reconfirms that it has a higher expansion rate. The higher axial velocity of the cough jet with the co-flow suggests that the airborne droplets and droplet nuclei will take shorter time to transport to a specified location in the axial direction compared to the cough jet without the co-flow.

The results are significant as they point to the adverse effect of increasing the infection risk that the co-flow could bring about. Since present study employs realistic airflow velocity, these findings could be useful to better design indoor spaces with minimal or no infection-risk. Our results further suggest that a cough should not be approximated as a steady jet. The work can be extended further by embedding particles of different sizes (corresponding to that ejected in a typical cough) in the flow and studying their dispersion with distance and time.

## Data Availability

The data that support the findings of this study are available from the corresponding author upon reasonable request.
